# The Iterative Convergent Design for Mobile Health Usability Testing: Mixed Methods Approach

**DOI:** 10.2196/11656

**Published:** 2019-04-26

**Authors:** Meshari F Alwashmi, John Hawboldt, Erin Davis, Michael D Fetters

**Affiliations:** 1 Memorial University St John's, NL Canada; 2 Department of Family Medicine University of Michigan Ann Arbor, MI United States

**Keywords:** mHealth, mixed methods, usability, eHealth, methods

## Abstract

Although patients express an interest in using mobile health (mHealth) interventions to manage their health and chronic conditions, many current mHealth interventions are difficult to use. Usability testing is critical for the success of novel mHealth interventions. Researchers recognize the utility of using qualitative and quantitative approaches for usability testing, but many mHealth researchers lack the awareness of integration approaches from advances in mixed methods research that can add value to mHealth technology.

As efficient usability testing proceeds iteratively, we introduce a novel mixed methods design developed specifically for mHealth researchers. The *iterative convergent mixed methods design* involves simultaneous qualitative and quantitative data collection and analysis that continues cyclically through multiple rounds of mixed methods data collection and analysis until the mHealth technology under evaluation is found to work to the satisfaction of the researcher. In cyclical iterations, early development is more qualitatively driven but progressively becomes more quantitatively driven. Using this design, mHealth researchers can leverage mixed methods integration procedures in the research question, data collection, data analysis, interpretation, and dissemination dimensions.

This study demonstrates how the iterative convergent mixed methods design provides a novel framework for generating unique insights into multifaceted phenomena impacting mHealth usability. Understanding these practices can help developers and researchers leverage the strengths of an integrated mixed methods design.

## Introduction

Published studies indicate that mobile health (mHealth) interventions are beneficial for patients across various diseases and age groups [1-4]. Academics and clinicians have an increasing interest in harnessing these mHealth interventions to improve health outcomes. Although patients express an interest in using mHealth to manage their health and chronic conditions, many current mHealth interventions are difficult to use [5]. Hence, developers of mHealth need efficient and effective approaches for development, but usability research methodology remains in a relatively nascent stage of development [6]. Usability testing is critical for the success of novel mHealth interventions. Although researchers recognize the utility of using qualitative and quantitative approaches for usability testing, many mHealth researchers lack the awareness of integration approaches from advances in mixed methods research (MMR) [7] that can add value to mHealth technology.

This paper advances the existing literature about the combined use of qualitative and quantitative research for mHealth by advancing a specific, integrated approach to mixed methods design appropriate to mHealth. When using qualitative and quantitative procedures without integration, researchers miss the opportunity for added value. Mixed methods methodologists express this as 1+1=2, as the quantitative and qualitative procedures are conducted as 2 independent studies with no particular synergy [8]. By using integrated procedures identified in the field of MMR, researchers can aspire for and achieve added value, as expressed by 1+1=3 [8,9].

The purpose of this paper is to articulate and illustrate the features of an iterative convergent mixed methods design. As efficient usability testing proceeds iteratively, we introduce a novel mixed methods design developed specifically for mHealth researchers. It offers a novel framework to generate unique insights into multifaceted phenomena related to mHealth usability. Understanding these practices can help developers and researchers leverage the strengths of an integrated mixed methods design.

### Background

#### Mobile Health

Effective health care strategies are required to ensure the right patient receives the right treatment at the right time. Advancements in mobile phones and tablets have led to the emergence of mHealth. The Global Observatory for eHealth of the World Health Organization defines mHealth as “medical and public health practice supported by mobile devices, such as mobile phones, patient monitoring devices, personal digital assistants, and other wireless devices” [10]. Recent advances allow seamless integration between smartphones and medical devices. This integration enables smartphones to store and analyze objective measurements such as heart rate, lung volume, and medication adherence. Advancements in machine learning and artificial intelligence have the potential to use these measurements, in combination with data collected via smartphones, to improve our understanding of disease etiology [11,12].

The significance of mHealth is highlighted by its ability to deliver timely care over distance to manage diseases. It is particularly important for rural areas with limited access to health care [13,14]. Moreover, mHealth strategies can enhance treatment outcomes while mitigating health care costs [15,16]. Hayes et al [16] illustrated why mHealth could reduce physician visits, resource consumption, and emergency room visits. The literature continues to evolve on applications of mHealth. For example, several published studies indicate that mHealth interventions are beneficial for patients across various diseases and age groups [1-4]. However, research on the development and usability methodology of such interventions remains in a relatively early stage.

#### Human-Centered Design

The International Organization for Standardization (ISO) 9241-210 standard defines human-centered design (HCD) as “an approach to systems design and development that aims to make interactive systems more usable by focusing on the use of the system and applying human factors/ergonomics and usability knowledge and techniques” [17]. The ISO uses the term HCD instead of user-centered design as it” addresses impacts on a number of stakeholders, not just those typically considered as users” [17]. However, in practice, these terms are often used synonymously.

**Figure 1:**
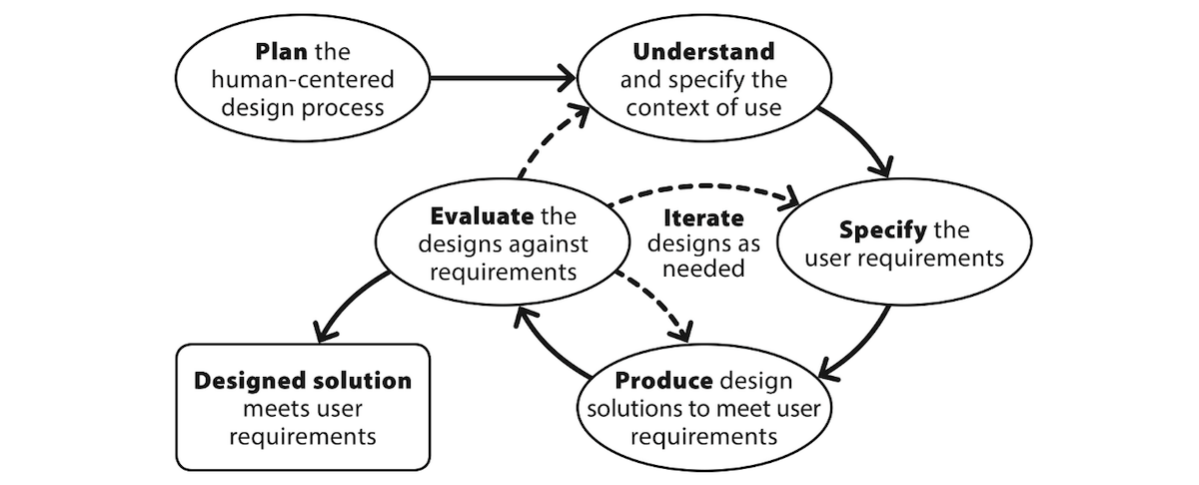
Human-centered design activity phases (ISO, 2010).

HCD has 4 defined activity phases: (1) identify the user and specify the context of use, (2) specify the user requirements, (3) produce design solutions, and (4) evaluate design solutions against requirements. The process model of HCD as defined in ISO 9241-210 is illustrated in Figure 1.

Researchers advocate for involving patients during development who are going to use the mHealth intervention to meet the patient’s needs and facilitate successful uptake. Testing mHealth interventions with patients reveals preferences and concerns unique to the tested population [5,18,19]. Developing an mHealth intervention with insights from stakeholders will potentially improve the process and outcome of mHealth interventions. The main goal of HCD is to increase the usability of mHealth technology.

This study offers an in-depth account of the HCD’s fourth activity phase, evaluate design solutions against requirements. This clarifies that this framework intends to focus on usability testing as one component of the more extensive design process.

#### Usability

The ISO defines usability as the extent to which a product can be used by specified users to achieve specified goals with effectiveness, efficiency, and satisfaction in a specified context of use [20]. Although this definition was published in 1998, it has been updated in 2018 without any changes to the core concepts. The definition is widespread and generalizable [21,22].

mHealth involves the interaction between multiple user groups through a system. As a result, the usability aspect is vital for the effective, efficient, and satisfactory use of mHealth interventions. Although patients express an interest in using mHealth to manage their health and chronic conditions, many mHealth interventions are not easy to use [5,23]. Difficulty in using an mHealth intervention may limit the user retention rate. A high dropout rate is one of the most significant barriers to mHealth adoption [24,25]. The majority of mHealth app publishers (83%) have less than 10,000 users who have used the app at least once a month [26]. These numbers are discouraging as according to a 2018 estimate, the average mHealth app costs $425,000 to develop [26]. By putting a more significant emphasis on usability, iterative improvements can reduce costs and enhance the long-term use and adoption of mHealth interventions [27-29].

Researchers recommend frequent and iterative usability testing to respond to users’ preferences, technical issues, and shortcomings [18,30,31]. It is also important to ensure that errors in understanding or using the intervention are addressed before testing the intervention in an efficacy trial [32]. A systematic review investigated the usability evaluation processes described in 22 studies related to mHealth applications [33]. The results suggest that the adoption of automated mechanisms could improve usability and stress the importance of adapting health applications to users’ need [33]. Including insights from key users of mHealth has the potential to improve the process and the outcome of the intervention [34].

Contemporary iterative development methods, such as prototyping, reduce the challenges that evolve during the development lifecycle [35]. Prototyping is defined as creating a simulation of the final mHealth technology that is used for testing before launch. Furthermore, researchers suggest including patients who are going to use the mHealth interventions to assist in the development of the intervention [5,18,19,33].

The ISO 9241-11 established usability standards [20]. These standards provide a measure of patients’ experienced usability. It focuses on effectiveness, efficiency, and satisfaction [20]. It is easier to quantify effectiveness and efficiency compared with satisfaction. Brooke developed the System Usability Scale (SUS) [36] and noted that “if there is an area in which it is possible to make more generalized assessments of usability, which could bear cross-system comparison, it is the area of subjective assessments of usability” [36]. Thus, the SUS was developed to quantify satisfaction (users’ subjective reactions to using the system) [36]. The SUS is an affordable and effective tool for assessing the usability of products [36]. It contains 10 statements that are answered on a 5-point Likert scale (Multimedia Appendix 1). Although this scale was developed in 1996, it is relevant and applicable to current research because it is short and easy to use. Many contemporary mHealth studies have been successful in combining both ISO and SUS to assess usability [37-39]. Table 1 describes the ISO 9241-11 usability constructs.

**Table 1:** Usability constructs and descriptions.

Constructs^a^	Metrics	Description
Effectiveness	Time to learn and use	Time to read the scenarios and to begin performing tasks
	Data entry time	Time to enter the data necessary for the execution of a task
	Tasks time	Time to accomplish given tasks
	Response time	Time of having the response to the requested information
	Time to install	Installation time of applications or its update
Efficiency	Number of errors	Number of errors made while reading scenarios and during the task execution
	Completion rate	The percentage of participants who correctly complete and achieve the goal of each task
Satisfaction	Usability score	The System Usability Questionnaire

^a^Adapted from Moumane et al [40].

#### Mixed Methods Research

MMR is gaining popularity and acceptance across disciplines and the world [41]. It draws from multiple scientific traditions and disciplinary backgrounds. MMR is defined as “the type of research in which a researcher or team of researchers combines elements of qualitative and quantitative research approaches for the broad purposes of breadth and depth of understanding and corroboration” [42]. MMR combines both closed-ended response data (quantitative) and open-ended personal data (qualitative) [41].

Although quantitative research historically has predominated in health sciences research, many contemporary phenomena in health care are difficult, if not impossible, to measure using quantitative methods alone [43]. The goal of qualitative research is to produce a deep understanding of a phenomenon. It can also be used to generate a hypothesis regarding a phenomenon, its precursors, and its consequences [44]. When the study phenomenon of interest is multifaceted and complex, a mixed methods approach is appropriate [43]. The National Institutes of Health best practices guideline and many mixed methods researchers advise distinguishing the quantitative purpose, the qualitative research questions, and the mixed methods questions [45]. Consequently, MMR can capitalize on the strengths of both methods, the depth of qualitative research and the breadth of quantitative research. The resulting mixed data can be integrated to balance the strengths and limitations of either method to provide a more comprehensive understanding under potentially complementary sources of evidence [43].

Understanding the principles and practices of integration is essential for leveraging the strengths of MMR. Fetters and Molina-Azorin [7] defined *integration* as the linking of qualitative and quantitative approaches and dimensions together to create a new whole or a more holistic understanding than achieved by either alone. Fetters et al examined vital integration principles and practices in MMR [46]. They provide approaches to integrating both research procedures and data in the design, methods, interpretation, and reporting dimensions of research [46]. Table 2 provides the relevant dimensions of MMR integration and illustrates how researchers can integrate those dimensions. These dimensions are relevant to mHealth, and additional information about MMR dimensions is explained elsewhere [7]. Through increasingly sophisticated approaches, MMR is viewed as an opportunity to address the highly complex, compelling, and even *wicked* research problems facing researchers in the health and social sciences [47]. Investigation of novel mHealth technologies is an important example of a highly complex research challenge that can benefit from a systematic mixed methods approach.

**Table 2:** Relevant dimensions of the mixed methods research integration.

Integration dimensions^a^	Mixed methods researchers integrate by
Rationale dimension	Citing a rationale for conducting an integrated mixed methods research study (eg, offsetting strengths and weaknesses, comparing, complementing or expanding, developing or building, and promoting social justice)
Study purpose, aims, and research questions dimension	Composing an overarching mixed methods research purpose and stating qualitative, quantitative, and mixed methods aims or multiple mixed methods aims with quantitative aims and qualitative questions
Research design dimension	Scaffolding the work in core (eg, convergent, exploratory sequential, and explanatory sequential), advanced (eg, intervention, case study, evaluation, and participatory), or emergent designs.
Sampling dimension	Sampling through the type, through the relationship of the sources of the qualitative and quantitative data (eg, identical sample, nested sample, separate samples, and multilevel samples), and through the timing (eg, same or different periods for collection of the qualitative and quantitative data)
Data collection dimension	Collecting both types of data with an intent relative to the mixed methods research procedures (eg, comparing, matching, diffracting, expanding, constructing a case, connecting, building, generating and validating a model, or embedding).
Data analysis dimension	Analyzing both types of data using intramethod analytics (eg, analyzing each type of data within the respective qualitative and quantitative methods and core integration analytics), using 1 or more core mixed methods analysis approach (eg, by following a thread, spiraling, and back-and-forth exchanges), or employing advanced mixed methods analysis (eg, qualitative to quantitative data transformation, quantitative to qualitative data transformation, creating joint displays, social network analysis, qualitative comparative analysis, repertory grid/other scale development techniques, geographic information systems mapping techniques, and iterative and longitudinal queries of the data).
Interpretation dimension	Interpreting the meaning of mixed findings (eg, where there are related data and drawing metainferences or conclusions based on interpreting the qualitative and quantitative findings) and examining for the fit of the 2 types of data (eg, confirmation, complementarity, expansion, or discordance). When the results conflict with each other, using procedures for handling the latter including reconciliation, initiation, bracketing, and exclusion.

^a^Adapted from Fetters and Molina-Azorin [7].

#### Importance of Mixed Methods in Usability Testing

Usability is a complex phenomenon. It is challenging to investigate usability comprehensively using only quantitative methods or qualitative methods in isolation, so-called *monomethod* approaches [6]. The alternative to using a monomethod approach is using diverse methods to generate a complete picture and reveal hidden patterns and novel relationships between variables and concepts [43]. To identify and resolve usability issues, various researchers emphasize the importance of using multiple methods and sources of data [18,48]. Although many studies collect both quantitative and qualitative data to test usability [49-51], mHealth researchers could benefit from advances being made for integration in mixed methods studies [46,52].

Despite the recognized and intuitive value of using mixed methods for mHealth usability testing, mixed methodologists have yet to articulate specific designs that guide the development and testing of mHealth interventions. A core MMR study design that is attractive for usability testing is the convergent design [52]. Also called by some authors as a concurrent parallel study [53] or, historically, a concurrent triangulation design [54], the convergent mixed methods design features the collection and analysis of both types of data and then merging of the data for the final interpretation [41].

##### The Iterative Convergent Mixed Methods Design

**Figure 2:**
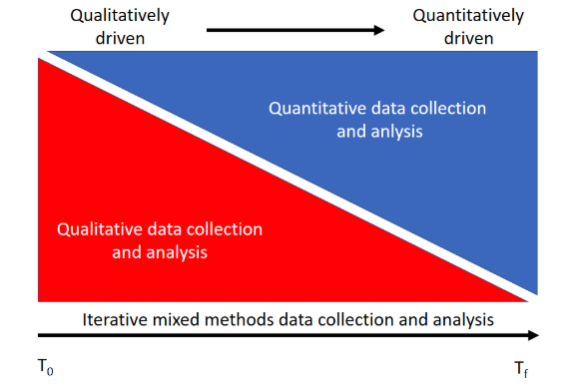
Evolution in an iterative convergent mixed methods design from qualitatively driven to quantitatively driven.

Owing to the iterative nature of usability testing, we propose a new variation of the convergent design specifically for mHealth, namely, the *iterative convergent mixed methods design*. We define an iterative convergent mixed methods design as an approach involving simultaneous qualitative and quantitative data collection and analysis that continues cyclically through multiple rounds of mixed methods data collection and analysis until the mHealth technology under evaluation is found to work to the satisfaction of the researcher. In cyclical iterations, early development is more qualitatively driven but progressively becomes more quantitatively driven; see Figure 2 [55]. Thus, the iterative convergent mixed methods design involves simultaneously collecting and analyzing qualitative and quantitative data and, as critically important, taking into consideration the iterative nature of mHealth technology development.

In the following, we articulate the features of an iterative convergent mixed methods design appropriate for mHealth intervention development and usability testing that incorporates an iterative process and is conducted according to the user’s health care and usability needs. Leveraging a specific mixed methods design can help fully integrate the 2 forms of data to enhance the understanding of the usability of mHealth interventions.

## Methodology

**Figure 3:**
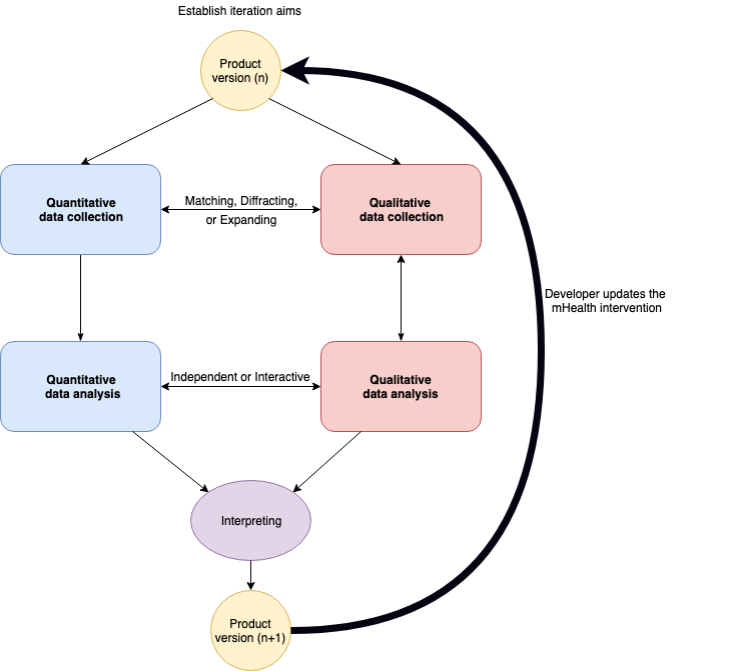
The iterative convergent mixed methods research design.

Fetters et al recommend considering the design, data collection procedures, interpretation, and analysis for achieving integration in a mixed methods study [46]. The iterative convergent design includes integration in various dimensions: the research aim/question, data collection, data analysis, and data interpretation. As illustrated in Figure 3, the results of each iteration inform further development of mHealth technology. These integration dimensions, as applied to usability testing, are discussed in more detail below.

### Step 1: Integration in the Research Aim/Question Dimension

An iterative convergent mixed methods design should have an MMR aim as well as specific quantitative research aims and qualitative research questions**.**

#### Mixed Methods Aim

The mixed methods aim is to illustrate, explore, and measure how to improve the usability of an mHealth intervention. A mixed methods aim should imply both qualitative and quantitative data collection methods. For example, illustrate and explore imply qualitative data collection, whereas measure implies qualitative data collection [7].

#### Quantitative Research Aims

Appropriate quantitative research aims include measuring effectiveness, efficiency, and satisfaction, as illustrated in Table 3. These constructs provide a measure of patients’ experienced usability. Effectiveness, efficiency, and satisfaction can be compared across iterations to identify the most usable mHealth technology.

#### Qualitative Research Questions

As illustrated in Table 3, appropriate qualitative research questions include clarifying and characterizing our understanding of mHealth intervention usability. Qualitative inquiry is particularly valuable for understanding how and why a phenomenon occurs, a theory explaining a phenomenon, or the nature of someone’s experience [56]. In usability testing, specific applications can include how and why participants make certain choices when using a prototype or their overall assessment of the utility. Usability testing may require or suggest a theory for its utility. The quality of the user’s experience is critical for an mHealth developer who is creating a desirable user-friendly system.

A recent study by Beatty et al [50] illustrates the mixed methods process as they collected both quantitative and qualitative data to determine the usability of a mobile app for technology-facilitated home cardiac rehabilitation. Quantitative data included the SUS and task completion rate, whereas the qualitative data included questions about the functionality of the mobile app [50].

**Table 3:** Matching of the construct’s quantitative variables and qualitative questions in a joint display depicting mixed methods of data collection.

Construct	Quantitative variables	Qualitative questions
Effectiveness	Time to learn and use	How did you learn to use the app? How can we reduce the time it takes to learn the app? What was your experience using the app? How can we reduce the time it takes to use the app?
	Data entry time	How can we reduce the time it takes to enter the data?
	Tasks time	How can we reduce the time it takes to complete the task?
	Response time	How do you feel about the app response time?
	Time to install	What are your thoughts about the time it took to install the app? The time it took to pair the medical device, if applicable?
Efficiency	Number of errors	What can we do to help users avoid the same error?
	Completion rate	What can we do to enhance the completion rate?
Satisfaction	Usability score	How often would you use the app? Why? Why not?; How do you feel about the complexity of the app?; How can we simplify it?; Do you have any recommendations to make the wording and interface easier to use? ; Would you need the support of a technical person to be able to use this system? How would you contact them: phone, email, or messaging? ; How did you find the integration of various functions in this app? How can we make it better?; How did you feel about the consistency of the app?; How can we simplify it?; Did you have any troubles when using the app? Where? How can we fix it? ; Did you feel confident when using the app? How can we make you more confident?; Did the app capture issues of importance to you?; Are there other ways to gather similar information?

### Step 2: Integration in the Data Collection Dimension

During usability testing, users will be asked to provide feedback optionally on paper and, later, on working prototypes. Testing usability with 5 participants will generally be sufficient for identifying significant issues for each version [57]. During each session, participants will be given specific tasks. Both quantitative and qualitative data will be collected during and after the completion of the tasks. Researchers have 3 key strategies for integration during data collection: matching, diffracting, and expanding.

#### Matching

The *matching* integration strategy involves intentionally asking qualitative questions that address the scales or constructs of quantitative instruments such that both instruments will elucidate data about the *same* concepts or domains [7]. For example, the constructs of both the ISO standards and SUS used during quantitative data collection can be matched with similar or related qualitative questions to generate related quantitative and qualitative data, as illustrated in Table 3. A mixed methods data collection joint display includes the major constructs of inquiry in the first column. The latter 2 columns include the quantitative data, for example, scales or items, and the qualitative data, for example, qualitative questions or observation types. For example, Beatty et al [50,58] used matching by integrating the task completion time (quantitative) with asking “I noticed that the _____ feature took you longer than some of the others. Tell me more about that?” (qualitative). They also expanded on the SUS by asking open-ended questions regarding the user’s experience with the mHealth intervention.

The qualitative questions in this table include both general and specific questions. Depending on the development needs, more general questions may be used initially, whereas later, more specific questions may be asked. When data become available, the same table structure can be populated with the findings; see *Step 4: Integration in the Data Interpretation Dimension*. The ordering of the columns is flexible according to specific project procedures.

#### Diffracting

The *diffracting* integration strategy involves intentionally asking qualitative questions that will address *different* aspects of the quantitative data, in the case of mHealth, the usability measure [58]. The intent is to obtain different *cuts of data* that will reveal information about different aspects of the usability that will not be addressed with the quantitative scales or items that are being collected [58]. Hence, for the ISO measures of effectiveness, efficiency, and satisfaction, qualitative questions might explore other facets, for example, animations, color patterns, sounds, and font size.

Diffracting can be used to address external factors to the user; such as the ease of connecting to the internet or connecting medical devices via Bluetooth. It is also important to develop an mHealth intervention that is energy efficient. mHealth interventions that require frequent charging of the smartphone or medical device are not recommended. Finally, developers should ensure that adequate resources are available to address medical and technical difficulties related to the mHealth intervention.

#### Expanding

The *expanding* integration strategy occurs when the findings from the 2 sources of data diverge and expand upon the phenomenon of interest by addressing both different aspects of a single phenomenon as well as a central phenomenon of interest [46]. Expansion involves intentionally asking qualitative questions that will be the same as the quantitative scales, while also measuring and asking qualitative questions that will address different aspects of usability. In essence, it reflects a hybrid strategy of using both matching, an area of overlap, and diffracting by looking at different aspects or facets of mHealth during the collection of data. Each of these integration strategies could be used effectively in usability testing.

##### Quantitative Data Collection

Current prototyping platforms, such as InVision and Adobe XD, integrate with Lookback to enable recording of the user’s interaction with a smartphone. These allow recording of the participant’s voice, nonverbal reactions, and mobile phone screen display. The researcher asks participants to complete a set of tasks and assess effectiveness, efficiency, and satisfaction. The researcher records the time to learn and use mHealth technology, data entry time, task completion time, response time, and time to install the mHealth technology. The researcher also records the number of errors and task completion rate. After completing the tasks, the researcher administers the SUS to assess the user’s satisfaction with the mHealth technology.

##### Qualitative Data Collection

The methods appropriate for assessment generally involve semistructured interviews during or after the participant’s use of the prototype. Researchers can utilize cognitive testing, also called cognitive interviewing [59-61]. The researcher asks participants to use the system while continuously thinking out loud as they move through the user interface [62]. Thinking aloud questions include “Tell me what you are thinking,” “What are you looking at?”, or “What’s on your mind?”. The goal is for the users to make their thoughts *transparent* to the researcher. Verbal probing is another alternative for eliciting additional information about mHealth technology. It is a more active form of data collection in which the cognitive interviewer administers a series of probe questions specifically designed to elicit detailed information beyond that which is typically provided by respondents [59].

Another alternative to these approaches involves a postuse debrief where the interviewer observes the user going through the mHealth intervention, notes decisions made, and, after use, enquires about decisions made along each step of the way. The strength of this approach is that the user can go through the version naturally without disruption as a real user would. However, the downside is the risk that the user may forget what specific thoughts or motivations influenced their decisions during real-time use. Postuse debrief questions may include (1) whether the tool captured issues of importance to the user, (2) whether the tool was easy to use and understand regarding question wording and interface, and (3) whether there were other ways the system could be improved.

A different option involves the collection of observations to record information about behavior. This can be done in real time through the collection of notes while observing or through recordings of the user’s interactions and using video elicitation interviews [63]. Video elicitation interviews question the user about their experiences and choices at certain points while interacting, for example, specific choices made and reasons for leaving a screen or returning to it. Tobii Pro can allow the researcher to track eye movements that convey behavioral patterns of use while interfacing with the mHealth technology.

Semistructured interviews and cognitive interviewing are suitable in the early stages of development. The goal is to identify *bugs* in the system, that is, anything that is dysfunctional or suboptimal. In later stages, verbal probing and video elicitation interviews are recommended to obtain specific data about the engineered changes.

### Step 3: Integration in the Data Analysis Dimension

There are 2 approaches for an integrated analysis: an interactive analysis strategy or an independent intramethod analysis [64].

#### Interactive Analysis Strategy

The *interactive analysis strategy* [64], also called a crossover-tracks analysis [65], means that the researcher considers the qualitative and quantitative findings, in real time, as the data are collected and analyzed. That is, the data are openly, actively, and interactively considered in the context of each other. Metaphorically, the data are *talking to each other*.

#### Independent Intramethod Strategy

The *independent intramethod strategy*, also called a parallel-tracks analysis [65], means that the researcher uses an intramethod (ie, within method) qualitative data analysis strategy separately or independently to the quantitative data analysis strategy. First, each type of data is examined using a strategy appropriate for the type of data, for example, statistical analysis of quantitative data and thematic analysis of the qualitative data. After the separate/independent analysis, the findings are then integrated to draw an overarching interpretation, so-called *metainferences* in MMR methodology [65].

For an iterative convergent design, the research can and likely will use both strategies depending on the stage of testing. The interactive analysis strategy is preferred, especially during early prototype testing when the number of users will invariably be smaller and there is an urgency for identifying major issues. As statistical analyses will not be feasible or necessary, this approach allows rapidly assessing user rankings of certain features, for example, using the SUS as well as their qualitative experiences with the system.

In later cycles of testing, the analysis may shift to a more independent intramethod analysis strategy. As a higher number of users engage and real-time automated digital user data emerge, the interactive analysis approach may become more challenging to conduct. Moreover, the independent intramethod analysis may be preferred when the scale of testing expands such that blinded quantitative data collection becomes more important. Doing so can enable the researcher to avoid validity threats to the data quality that could occur by changing the data collection approach or by sharing patterns with users in real time. For example, Kron et al [66] linked the qualitative findings from learners’ reflections on their experiences after completing the qualitative and quantitative analyses.

#### Combined Independent and Interactive Data Analysis

The third option can involve an iteration of both interactive and independent data analyses, that is, user survey and interview data conducted in real time can be looked at interactively, whereas automated data collection that accumulates as the number of users expands may be brought into the results of the interactive analysis after being examined independently. The exact approach may vary and evolve according to development needs.

#### The Fit of the 2 Types of Data When Considered Together

Comparing both the qualitative and quantitative findings allows researchers to examine the similarities, differences, or contradictions. This comparison also allows researchers to obtain an expanded understanding of when the qualitative and quantitative findings from the analyses are merged for an interpretation. Similarities occur when there is *convergence* or confirmation between the qualitative and quantitative findings. Differences occur when the 2 types of data illustrate different, nonconflicting interpretations, so-called *complementarity* [67]. There is an expanded understanding, namely, *expansion,* when qualitative and quantitative finding provides a broader understanding of a central commonality [7,46]. Contradictions occur when there is *discordance* or divergence between the findings of the qualitative and quantitative data. To handle discordance, Fetters et al recommend gathering additional data, reanalyzing existing databases to resolve differences, seeking explanations from theory, or challenging the validity of the constructs [7,46].

### Step 4: Integration in the Data Interpretation Dimension

A key challenge in mixed methods studies is how to merge the qualitative and quantitative data. A very promising approach of growing popularity among mixed methods researchers is the creation of a joint display [68]. Table 4 provides an example of presenting matched quantitative and qualitative data through a joint display. This joint display is derived from a randomized multisite mixed methods trial designed to compare a medical student’s attitudes and experiences regarding the intervention, a virtual human-computer simulation program teaching communication skills, or a control, a computer-based learning module focused on teaching communication skills [66]. The data collection for this project included usability-focused questioning [8].

Joint displays allow researchers to integrate data through visual means to draw out new insights beyond the information gained from the separate quantitative and qualitative results [46,68]. Joint displays are commonly built by organizing quantitative and qualitative findings of a related construct or topic in a table. When matching has been used during data collection, this process follows naturally. In the case of mHealth, the joint display might include the usability constructs, user’s perceptions, and an image or even a video representing the task [69]. For example, SUS and ISO metrics can be used to populate the numerical score and SD in the quantitative column. In addition, themes and representative quotations can be used to populate the qualitative column. In the final column, metainferences, an interpretation in consideration of the qualitative and quantitative findings, are made about the findings when analyzed together.

### Step 5: Developer Updates the Mobile Health Intervention

After merging the data and drawing interpretations about their cumulative meaning (making metainferences), an iterative convergent mixed methods design then involves the results being communicated to the developers who will include the recommendations in the new iteration of the intervention. Moreover, as the developers make changes, they may also have specific questions to be answered in the subsequent cycle of iterative convergent data collection. Thus, newly emerging questions are added into subsequent rounds of data collection. In general, both qualitative and quantitative data (task completion rate, task completion time, number of errors, completing rate, and the SUS questionnaire) should be compared with each iteration for new mHealth versions.

### Step 6: Iterative Evaluation

As illustrated in Figure 3, after 1 cycle of iterative evaluation, the next step is to develop a new version that has incorporated the findings from the previous evaluation. For example, Beatty et al [50] compared the task completion success rate and SUS across iterations of the mHealth intervention. The procedure will be similar by going back to *Step 2: Integration in the Data Collection Dimension*. With the new iteration, there will be new questions to ask, sometimes more general and sometimes more specific, depending on what changes were made. Furthermore, a new iteration is also required when the researcher introduces a new feature or functionality to the mHealth intervention.

On the basis of the results of the usability test, many changes may be required. The researcher should prioritize these changes while focusing on the user’s needs. Generally, the magnitude of data collection and intensity will change. In the early rounds of development, the qualitative component of the mixed methods evaluation will weigh more heavily for identifying the macrolevel changes (Figure 1). This is more of a qualitatively driven mixed methods approach [64]. In subsequent iterations, as the prototype moves from paper to digital prototype to product, changes may depend much more heavily on the quantitative automated analyses that can accumulate with increased numbers of users, a quantitatively driven approach. Hence, in later cycles, the quantitative data may help identify problems, whereas the qualitative data can be used to identify solutions.

### Reaching Closure in an Iterative Convergent Mixed Methods Design

Many researchers use the concept of saturation when conducting usability testing [70,71]. Saturation represents the point at which the researcher stops collecting data based on the criterion of not finding new information relevant to the development of the mHealth application. Researchers also have the option for longitudinal evaluation by comparing user satisfaction with the new mHealth technology with the result of the SUS across iterations. Similarly, qualitative data about specific features can be compared as well. After usability testing, the final prototype of the mHealth intervention can then be included in a pilot study for final refinement before launching it in a larger trial. During these subsequent phases, the iterative convergent mixed methods design will naturally continue, even into the trial.

**Table 4:** A joint display adapted from Kron et al’s MPathic-VR mixed methods trial comparing a virtual human simulation and a computer-based communications module that illustrates medical students’ attitudes and experiences in both trial arms.

Domains	MPathic-VR		Computer Based Learning		Interpretation of mixed methods findings
	Attitudinal item, mean (SD)	Qualitative reflection; illustrative quotes	Attitudinal item, mean (SD)	Qualitative reflection; illustrative quotes	
Verbal communication	5.02 (1.62)	“How to introduce myself without making assumptions about the cultural background of the patient and the family”	3.89 (1.67)	“This educational module was useful for clarifying the use of SBAR and addressing ways that all members of a health care team can improve patient care through better communication skills”	Intervention arm comments suggest deeper understanding of the content than teaching using memorization and mnemonics as in the control, a difference confirmed by higher attitudinal scores
Nonverbal communication	4.11 (1.85)	“Effective communication involves non-verbal facial expression like smiling and head nodding”	2.77 (1.45)	None	Intervention arm comments address the value of learning nonverbal communication, the difference confirmed by attitudinal scores
Training was engaging	5.43 (1.55)	“Reviewing the video review was a great way to see my facial expressions and it allowed me to improve on these skills the second time around”	3.69 (1.62)	“This experience can be improved by incorporating more active participation. For example, there could have been a scenario in which we would have to select the appropriate hand-off information per SBAR guideline”	Intervention arm comments reflect engagement through the after-action review, whereas the control comments suggested the need for interaction, the difference confirmed by higher attitudinal scores
Effectiveness in learning to handle emotionally charged situations	5.13 (1.48)	“I tend to try to smile more often than not in emotionally charged situations and that may result in conveying the wrong message”	2.34 (1.35)	“I anticipate that high-stress situations where time is exceedingly crucial requires modification to the methods presented.”	Intervention arm comments indicate awareness of communication in emotionally charged situations, yet control comments indicate the need for additional training, a difference confirmed in attitudinal scores

## Discussion

Here, we emphasize the need and process for mHealth researchers to use state-of-the-art mixed methods procedures. Previous single method usability studies were limited in their findings. Some studies have assessed usability using only qualitative data [72-74]. These studies can only elucidate an understanding of how and why participants make certain choices when using a prototype or their overall assessment of the utility. On the contrary, some studies have used quantitative data exclusively to assess usability [75,76]. These studies are limited to specific questions about usability and could miss valuable experiential information.

### Features of the Iterative Convergent Mixed Methods Design

Despite the recognized value of using mixed methods for usability testing [49-51,77], researchers have lacked a specific design and clear procedures featuring an integrated approach that is focused on mHealth development. We believe, and the identified literature supports, that many researchers in the field are only using qualitative and quantitative procedures separately without a focus on the features of integration [37,50,51]. This illustrates explicitly why mHealth intervention researchers need a better understanding of how to incorporate the latest advances in MMR methodology, which explicitly emphasizes integration, and has procedures for achieving it.

We suggest the following criteria for evaluating the quality of studies that have used the iterative convergent mixed methods design:

1. The authors report on an empirical mHealth-related usability study.
2. The authors use an integrated mixed methods approach, defined as the collection, analysis, and integration of quantitative and qualitative data [52].
3. The authors compare the results of various iterations of the mHealth intervention.

The iterative convergent mixed methods design provides a clear framework for integrating quantitative and qualitative data to assess usability. As illustrated in Figure 3, there are multiple dimensions during testing, questions, data collection, analysis, and interpretation as well as subsequent data collection that characterize an iterative convergent mixed methods design.

With this design, researchers will start with a mockup, prototype, or the actual mHealth intervention that is represented in the diagram by the circle named the mHealth technology version. In each round, the researcher will evaluate aspects of the version using both qualitative and quantitative research aims and, importantly, making overarching interpretations or metainferences based on the findings of both types of data that inform the next steps [65]. During data collection, the researcher can use matching, diffracting, or expanding as data are collected. Employing specific data collection approaches, the constructs explored quantitatively with scales can be explored with a similar line of qualitative questioning or inquiry. Once the data are brought together, they are compared so as to examine their confirmation, expansion, differing interpretations, or discordance [46]. As successive versions of the mHealth technology are produced, each version will involve both qualitative and quantitative data collection brought together for an integrated analysis. Iterative qualitative and quantitative data collection can be compared with each iteration to create the most usable version of the mHealth technology.

### Limitations

There are potential limitations to the current usability approach. Although the small sample size may resolve the majority of usability issues [57], usability testing with a small number of individuals will generally reveal major flaws or *bugs* in the system. As the mHealth intervention becomes refined and moves from the protocol stage to the actual use stage, access to quantitative data rapidly increases and becomes more of a focus.

Usability testing can be conducted on the Web or in a laboratory setting. The value of Web-based testing is that users can participate from their natural context and use their own devices. It is also more cost-effective, and users can be in any location with an internet connection. In a laboratory setting, the researcher can probe users while they walk through their tasks, gather visual cues, assist stumped users, and ask new questions during the testing session.

We acknowledge that there are other methods, including other mixed methods designs [46], potentially applicable for usability and design research, for example, mixed methods interventions or trials. There are also other scales that can be used to quantify the satisfaction construct, such as the Post Study System Usability Questionnaire [78]. Addressing all of these methods and scales extends beyond the scope of our current focus.

### Conclusions

A usable mHealth intervention with high user satisfaction can have a significantly positive effect on mHealth adoption, resulting in improved health outcomes and quality of life and reduced overall health care costs. Effective mHealth interventions are critically important for empowering patients to manage their health and also potentially enable them to participate more actively in shared decision making with their health care providers. This study offers a novel framework to guide mHealth research that has the potential to generate unique insights into multifaceted phenomena related to usability. Understanding these practices can help developers and researchers leverage the strengths of an integrated mixed methods design.

## Appendix

Multimedia Appendix 1 System Usability Scale.

## Abbreviations

HCD: human-centered design
ISO: International Organization for Standardization
mHealth: mobile health
MMR: mixed methods research
SUS: System Usability Scale
