# Supplementary material for: The Iterative Convergent Design for Mobile Health Usability Testing: Mixed Methods Approach
Source: JMIR Mhealth Uhealth. 2019 Apr 26;7(4):e11656. doi: 10.2196/11656 (PMC6658163; doi:10.2196/11656)
Supplement: Multimedia Appendix 1 [file mhealth_v7i4e11656_app1.docx]

## Appendix 1 – System Usability Scale

Participants are asked to score the following ten items with one of five responses that range from Strongly Agree to Strongly disagree (Brooke, 1996):

1. I think that I would like to use this system frequently.
2. I found the system unnecessarily complex.
3. I thought the system was easy to use.
4. I think that I would need the support of a technical person to be able to use this system.
5. I found the various functions in this system were well integrated.
6. I thought there was too much inconsistency in this system.
7. I would imagine that most people would learn to use this system very quickly.
8. I found the system very cumbersome to use.
9. I felt very confident using the system.
10. I needed to learn a lot of things before I could get going with this system.
